# Combining Dipole and Loop Coil Elements for 7 T Magnetic Resonance Studies of the Human Calf Muscle

**DOI:** 10.3390/s24113309

**Published:** 2024-05-22

**Authors:** Veronika Cap, Vasco Rafael Rocha dos Santos, Kostiantyn Repnin, David Červený, Elmar Laistler, Martin Meyerspeer, Roberta Frass-Kriegl

**Affiliations:** 1 High Field MR Center, Center for Medical Physics and Biomedical Engineering, Medical University of Vienna, 1090 Vienna, Austria; 2 Institute for Clinical and Experimental Medicine, 140 21 Prague, Czech Republic; 3 Institute of Biophysics and Informatics, First Faculty of Medicine, Charles University, 121 08 Prague, Czech Republic

**Keywords:** magnetic resonance imaging, magnetic resonance spectroscopy, radiofrequency coil, ultra-high field, muscle metabolism

## Abstract

Combining proton and phosphorus magnetic resonance spectroscopy offers a unique opportunity to study the oxidative and glycolytic components of metabolism in working muscle. This paper presents a 7 T proton calf coil design that combines dipole and loop elements to achieve the high performance necessary for detecting metabolites with low abundance and restricted visibility, specifically lactate, while including the option of adding a phosphorus array. We investigated the transmit, receive, and parallel imaging performance of three transceiver dipoles with six pair-wise overlap-decoupled standard or twisted pair receive-only coils. With a higher SNR and more efficient transmission decoupling, standard loops outperformed twisted pair coils. The dipoles with standard loops provided a four-fold-higher image SNR than a multinuclear reference coil comprising two proton channels and 32% more than a commercially available 28-channel proton knee coil. The setup enabled up to three-fold acceleration in the right–left direction, with acceptable g-factors and no visible aliasing artefacts. Spectroscopic phantom measurements revealed a higher spectral SNR for lactate with the developed setup than with either reference coil and fewer restrictions in voxel placement due to improved transmit homogeneity. This paper presents a new use case for dipoles and highlights their advantages for the integration in multinuclear calf coils.

## 1. Introduction

Magnetic resonance spectroscopy (MRS) has been extensively used to study energy metabolism in working muscle, such as the human calf [1,2,3,4]. ^1^H MRS [5] and MRI can provide important information complementing data on oxidative metabolism derived from phosphorous-31 (^31^P) MRS. Examples of metabolites that can be targeted with proton MRS are intramyocellular lipids, acetylcarnitine [6,7], carnosine, taurine, total creatine [8], and deoxymyoglobin [9]. Some resonances undergo a modulation of visibility as a function of muscle fiber orientation like the CH_2_ resonance of creatine, which also reflects PCr dynamics [10], or lactate, which is detectable after intense exercise using sequences that selectively suppress the lipid signal resonating at the same frequency as lactate’s CH_3_ group [11,12]. Furthermore, muscle functional MRI (mfMRI) is sensitive to the metabolic and hemodynamic processes of muscle activation, resulting in *T*_2_ and signal intensity changes [13] that convey quantitative information on the spatial distribution of muscle recruitment [14]. Metabolic and functional information can be complemented with perfusion MRI [15,16], indicative of oxygen and substrate supply to maintain metabolic processes. Diffusion-tensor imaging (DTI) can deliver structural information [17,18], and strain (i.e., the displacement of tissue) can be imaged via phase-contrast or spatial-tagging MRI [19].

The motivation of the presented work was primarily to study energy metabolism in exercising triceps surae muscles, i.e., gastrocnemius lateralis, gastrocnemius medialis, and soleus. While oxidative processes can be investigated to a large extent using ^31^P MRS, anaerobic mechanisms, which are difficult to capture using ^31^P MRS, become increasingly dominant at higher exercise intensities. Since these involve lactate production, a metabolite accessible through proton (^1^H) MRS, combined with ^1^H/^31^P MRS, would allow for more comprehensive studies of muscle physiology by capturing both oxidative and glycolytic metabolic processes [11]. This application requires high measurement sensitivity for both nuclei. The signal-to-noise ratio (SNR) can be increased by moving to ultra-high field, but optimized radiofrequency coils are needed to fully capitalize on this benefit [20]. The advanced ^1^H MRS and MRI techniques mentioned above, as well as X-nucleus spectroscopy, set demanding requirements for RF coil performance including excellent coverage of the targeted muscles with a homogeneously high SNR and transmit efficiency in terms of power and specific absorption rate (SAR), as well as parallel imaging capabilities. 

Since the goal was to study a dynamic process over time, using two separate coils that are exchanged between ^1^H and ^31^P measurements was not an option, and a combined coil for both nuclei was needed. Several approaches for designing multinuclear coils have been developed over the past decades, each having different advantages and drawbacks [21]. One method involves using the same physical conductor structure for both nuclei by making it resonant at both frequencies, traditionally through a frequency splitting trap. The additional component losses lead to about 25 to 30% loss in SNR, with trade-offs between the two frequencies depending on the trap values chosen [22,23]. Alternatively, switching between the two frequencies using a PIN diode is also a possibility, but this leads to ~35% SNR loss for one nucleus, and the reverse breakdown voltage of PIN diodes limits the power for the other [24,25]. These approaches are well suited for many X-nucleus applications, where the ^1^H part of the coil is mainly used for localization and shimming, and some ^1^H performance can be sacrificed in favor of X-nucleus sensitivity. However, excellent ^1^H performance was required in the metabolic studies envisioned here. Therefore, a multistructure approach with separate arrays for the two nuclei was favored. The primary goal of this work was to develop a ^1^H coil for 7 T, with very high transmit (Tx) and receive (Rx) performance in the gastrocnemius and soleus muscles of the human calf, which can accommodate an additional, highly sensitive ^31^P array with minimal compromises in ^1^H performance.

To satisfy these requirements, an array of dipole elements was investigated for ^1^H excitation and reception. While dipoles are typically used for deeper targets such as torso [26,27] and brain [28], we also see multiple benefits for the application in the calf. Firstly, using dipoles circumvents the inherent B_1_^+^ inhomogeneity of loop coils at ultra-high field, which can only be partly compensated by phase shimming and thus yields a more homogeneous excitation in the region of interest (ROI). Furthermore, the intrinsic orthogonality in B_1_ fields between dipoles and loops provides the possibility of (1) adding an array of ^1^H Rx-only loops that are geometrically decoupled from the dipoles and (2) limiting unwanted interactions to a prospective ^31^P loop array. Combining transceive (TxRx) dipoles with Rx-only loops has been used to increase the SNR in recent 7 T cardiac [27] and head coils [29,30,31]. While these works focused particularly on increasing the central SNR, which was not a primary concern for this application, we hypothesized that the approach would still be beneficial, as it enables a stacked arrangement of receivers. This increases the number of Rx elements without inducing excessive noise correlation, in turn improving the Rx performance of the overall setup, particularly with respect to parallel imaging [32,33]. 

The envisioned ^1^H coil design consists of two nested layers, with a prospective ^31^P array added as a third layer in the future. Managing spatial constraints and interelement crosstalk in such a high-density setup is challenging. Twisted pair coils are a recently proposed type of flexible transmission line resonator that has fewer lumped elements and has been speculated to provide better mutual decoupling than standard coils [34,35]. Additionally, while standard Rx-only coils require active detuning networks with a relatively large inductor for transmission decoupling, twisted pair Rx-only coils can be detuned with a simpler broadband mechanism [36]. Using twisted pair coils instead of standard loops could avoid potential interactions between the trap inductances and dipoles, reduce coupling issues between the ^1^H Rx elements and ^31^P loops, and save valuable space in the coil housing.

In this study, the ^1^H Tx, Rx, and parallel imaging performance of an array of three dipoles with and without an array of six Rx-only standard loop or twisted pair coils was investigated. The results were compared to those of a ^31^P-optimized custom ^31^P/^1^H calf coil previously developed by our group [37] and a commercial 28-channel ^1^H-only knee coil (QED Knee Coil, distributed via Siemens Healthineers, Erlangen, Germany). 

## 2. Materials and Methods

### 2.1. Coil Design and Implementation

**Figure 1:**
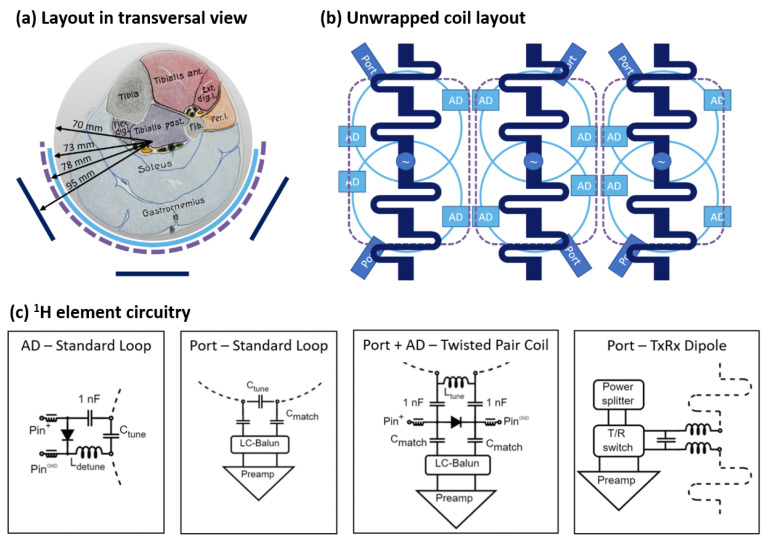
(**a**) Transversal view of the coil layout and phantom with an anatomical sketch of the region of interest. (**b**) Unwrapped coil layout with the ^1^H standard receive loops (light blue), ^1^H TxRx dipoles (dark blue), and prospective ^31^P TxRx array (purple dashed outline). Note that the twisted pair coils were placed in the same arrangement as the standard loops shown but did not include a separate active detuning network (AD). (**c**) Circuitry and interfacing for all investigated coil elements.

The proposed coil design features a half-cylindrical geometry covering the muscles of interest located in the human calf, i.e., gastrocnemius and soleus or triceps surae. This geometry not only enables the easy and comfortable positioning of the leg but also ensures good coil loading for a variety of subject sizes, as the calf rests in the coil former, molding to its shape. The placement of the ^1^H coil elements in two nested layers is shown in Figure 1a. The outermost layer of the coil is an array of three meander-shortened dipoles (*l* = 150 mm) based on a design by Raaijmakers et al. [26] used for ^1^H transmission and reception. A three-dipole layout is preferred over a design with a higher element count, as it can be operated without decoupling circuitry [38,39], due to the larger spacing between the individual dipoles. This improves the robustness and reduces the complexity of the overall high-density coil setup. To maximize ^1^H Rx sensitivity, an array of six ^1^H Rx-only loop coils (*d* = 70 mm) is placed closest to the ROI. The elements are arranged in two rows, with pairs of overlapping loops underneath each dipole for geometric decoupling, as shown in Figure 1b. A three-element ^31^P TxRx loop array can be added between the two ^1^H arrays in the future, as indicated by the dashed purple line in Figure 1. 

The focus of this work was on the development and performance evaluation of the ^1^H array. To this end, three different setups were implemented for a detailed investigation and comparison through bench and MR measurements: (1) the dipole array alone, (2) the dipole array with six standard Rx-only loops, and (3) the dipole array with six Rx-only twisted pair coils. 

The dipoles were fabricated as printed circuit boards and mounted on 3D-printed holders providing mechanical stability. The standard Rx loops were made from copper wire with a diameter of 1 mm. Each loop included two active detuning (AD) traps for transmission decoupling. The traps were arranged transversally (see Figure 1b), as shown to be favorable by Avdievic et al. [30]. 

The twisted pair coils were assembled from two PTFE-insulated wires (22 AWG, stranded silver-plated copper 19/0.15 mm, RS Components, Gmuend, Austria) that were manually twisted and bent into a circular shape, so that their ends met at opposite sides of the coil. This made the coil self-resonant at approximately 250 MHz. To operate the coil, one of the gaps was used as a coil port and an inductor inserted to tune it to the Larmor frequency. The other gap was left open and secured with a small piece of shrink tubing. A broadband active detuning network was used for transmission decoupling, which used a PIN diode to short the coil port during transmission [36]. All Rx-only elements were preamplifier-decoupled [40], which was adjusted by adapting the cable length between the loop and a low-noise preamplifier (0.5 dB noise figure, 27.2 ± 0.2 dB gain, Siemens Healthineers, Erlangen, Germany) to provide the necessary phase shift at the Larmor frequency. Schematic drawings of tuning, matching, and detuning circuits for the ^1^H elements are summarized in Figure 1c. 

Both types of Rx loops were attached to a 2 mm thin plastic former placed directly on the cylinder-shaped phantoms (*d* = 14 cm, *h* = 20 cm) used in the bench and MR measurements. For the bench and MR imaging tests, a phantom cylinder was filled with gel produced according to the ASTM F2182-11a standard [41], containing deionized water, 30 mM KH_2_PO_4_, 10 g/L methacrylic acid to reduce convection, and 1 mL/L Gd-based contrast agent to reduce *T*_1_. For MR spectroscopy measurements, a phantom containing ~71 mM of lactate in aqueous solution, conductivity-matched to the gel phantom used in imaging experiments, was prepared.

The transmit coil interface included a 3-way Wilkinson power divider as well as one transmit/receive switch and low-noise preamplifier per dipole. The latter were constructed using PIN diode switches and two-stage lumped element quarter-wave impedance transformation networks. Static B_1_^+^ shimming was optimized in a simulation following the workflow described in Section 2.4. The determined optimum was implemented by varying the cable lengths from the coil interface to the respective dipoles.

### 2.2. Bench Measurements

Tuning, matching, and interelement coupling were evaluated through transmission and reflection S-parameter measurements on a vector network analyzer (E5071C, Keysight Technologies, Santa Rosa, CA, USA), with all elements tuned but not connected to low-noise preamplifiers. The dual-loop probe method [42] was used to characterize the Rx coils’ quality factors. The unloaded vs. loaded quality factors (*Q* ratio) were measured for a single standard loop and twisted pair coil with tuning and detuning circuits but without a matching network. The active detuning and preamplifier decoupling performance was determined with the single-loop probe method [43] via *S*_11_ difference (Δ*S*_11_) measurements at the Larmor frequency. For the active detuning efficiency, the Δ*S*_11_ between the tuned and detuned state of the Rx coils was measured. The Δ*S*_11_ between the coils with preamplifiers plugged (and powered at 10 V) and unplugged (and the coils 50 Ω-terminated instead) was measured to evaluate preamplifier decoupling. 

The insertion loss of the system plug, coaxial cables, and the dipole interface (power splitter and transmit/receive switch) was measured via the transmission between the Tx connector in the system plug to each of the output channels including a coaxial cable of the same type and similar length as used to connect the dipoles. 

### 2.3. MR Measurements

All MR measurements were performed on a whole-body Magnetom Terra dot Plus 7 T (Siemens Healthineers, Erlangen, Germany) MR scanner. The protocol described in detail below was repeated with the same sequence parameters and slice positions for the three developed setups as well as with two reference coils. The first reference coil (“multinuclear reference”) was a two-channel ^1^H, three-channel ^31^P transceiver coil designed for calf muscle studies [37]. It features a similar half-cylindrical geometry (*d*_inner_ = 140 mm) as the coil developed in this work, with two nested layers of loop arrays for the two nuclei. The ^1^H part of the coil is the outer layer (*r* = 84.5), which is formed by two ^1^H TxRx loops, each 125 × 125 mm^2^ in size, with a shared conductor in between. As a second reference coil (“^1^H-only reference”), a state-of-the-art commercially available ^1^H-only extremity coil (QED Knee Coil, distributed via Siemens Healthineers, Erlangen, Germany) was chosen. This reference consists of a circularly polarized birdcage coil for transmission and 28 Rx elements arranged in three rings, fully surrounding a cylindrical opening (*d*_inner_ = 154 mm). 

Data processing and visualization were performed in MATLAB R2021a (The MathWorks Inc., Natick, MA, USA). For the evaluation and display of the results, a representative transversal slice across one row of Rx loops was chosen. Quantitative values (i.e., normalized B_1_^+^, SNR and g-factors) were calculated for a semicircular ROI representing roughly the gastrocnemius and soleus muscles of the human calf. 

B_1_^+^ maps were acquired by measuring flip angle maps [44] and scaling to 1 W input power. Boxplots were used to visualize the distribution of voxel-wise B_1_^+^ values across the ROI, with the median and interquartile range serving as measures of transmit field strength and homogeneity. To evaluate Rx performance, a noise-only scan (no radiofrequency excitation or spatial encoding gradients) was acquired, from which the noise correlation matrix was calculated. The SNR was calculated from a gradient echo (GRE) scan (TR/TE = 9.2/4.24 ms, FOV = 156 mm × 156 mm, 1 mm nominal in-plane resolution, 4 mm slice thickness) using the pseudo-multiple-replica method [45,46] with *n* = 200 replicas. To investigate the coil sensitivity at different distances to the coil, SNR profiles were calculated as radial averages from the phantom’s surface to its center. The parallel imaging performance was investigated by generating accelerated images and g-factor maps from the noise correlation matrix and raw GRE data, for 2-, 3-, and 4-fold GRAPPA [46] acceleration in the left–right direction. The g maps were smoothed with a 2D Gaussian kernel (SD = 1.5 mm), and the mean and maximum g-factors were calculated. 

To investigate the developed coil’s advantage for the intended lactate measurements, the best-performing setup and the two reference coils were used to acquire lactate spectra on the spectroscopy phantom (~71 mM lactate in aqueous solution). The measured voxel (70 × 20 × 50 mm^3^) was placed in a position consistent with the MRS of the gastrocnemius muscle in vivo (left leg, feet forward, supine position). The spectra were acquired in a single-shot approach using a semi-LASER sequence [47], with TE = 20 ms and an acquisition vector size of 1024 points (spectral width = 2500 Hz). Postprocessing was performed using an in-house Python script, where each channel was phased to the lactate methyl signal at 1.3 ppm. The SNR was calculated for each channel, from the maximum lactate signal and the standard deviation of 100 points from a noise-only region (index 100 to 200 of the FFT FID) [48]. The channels were then combined through a weighted average based on their individual SNRs. For visualization, the measured lactate spectra were line-broadened (3 Hz), zero-filled to 2048 points, and scaled to SNR. 

Additionally, the best-performing newly developed setup was also used to acquire an anatomical GRE image of a pork leg to evaluate the coil’s imaging performance on a biological sample with a similar cross-section as a human calf.

### 2.4. Electromagnetic Simulations

Simulations were used at two stages in the coil development process. First, static B_1_^+^ shimming was optimized for the dipole array on a phantom (relative permittivity *ε*_r_ = 75, conductivity *σ* = 0.59 S/m), by varying the phase shift in 10° steps to minimize peak SAR and maximize B_1_^+^ strength and homogeneity. Second, simulations were also used to evaluate B_1_^+^ and specific absorption rate (SAR) of the dipole array with standard Rx loops on the phantom for comparison to the MR results and on a human voxel model (Ella from the Virtual Family [49]). Since the model’s calf is flattened in the back, the resulting gap was filled with skin to ensure a more realistic filling of the coil.

Simulations were performed using full-wave 3D electromagnetic simulation and circuit co-simulation [50,51]. For this, the coil geometries were modeled in XFdtd (Remcom, State College, PA, USA); lumped elements were replaced by 50 Ω voltage sources to efficiently calculate electric and magnetic fields via the finite-difference time-domain (FDTD) method. Advanced Design System (Keysight Technologies, Santa Rosa, CA, USA) was then used to model tuning and matching circuits. The simulation results were combined and analyzed in MATLAB. A dedicated in-house toolbox (SimOpTx, Center for Medical Physics and Biomedical Engineering, Medical University of Vienna, Austria) employing the quadratic form power correlation matrix formalism [52,53] was used to calculate the specific absorption rate (SAR) per 10 g of tissue. Since the coil interface and cabling were not included in the simulation, the associated losses were accounted for by a scaling factor approximated from the bench measurements described above. B_1_^+^ maps were generated from the simulated data for a slice corresponding to the one chosen in the measurements. SAR was displayed as transversal maximum intensity projections (SAR MIPs). 

## 3. Results and Discussion

### 3.1. Bench Measurements

All elements were tuned to the Larmor frequency and matched to −11.9 dB or better. The worst coupling between two elements before preamplifier decoupling was −19.9 dB for the dipole array, −14.7 dB for the standard Rx loop setup, and −14.3 dB for the twisted pair coil setup. For the latter two, the highest interelement coupling was measured between neighboring, nonoverlapping loops. The *Q* ratio was 130/7 = 18.6 for the standard Rx loops and 104/14 = 7.4 for the twisted pair coils, showing that both coil types were sample-noise-dominated. The bench evaluation of transmission decoupling (comparing tuned and detuned states) yielded a Δ*S*_11_ > 30 dB (reaching the noise floor of the single-loop probe measurement) for standard loops and Δ*S*_11_ ≈ 18 dB for twisted pair coils. For preamplifier decoupling (coils connected to low-noise preamplifier versus 50 Ω termination), Δ*S*_11_ was approximately 20 dB for standard loops as well as twisted pair coils. Compared to the standard loops, the twisted pair coils were less sensitive to element placement, making the array quicker to assemble. For the coil interface, bench measurements revealed a 2.6 dB insertion loss including the three-way power splitter, transmit/receive switches, cables, connectors, and well as the system plug. 

### 3.2. Transmit Performance

The phases for static B_1_^+^ shimming for the dipole array were optimized as [0°, 100°, 200°]. Only minor differences in B_1_^+^ and SAR were observed, with up to ±20° variation from this setting in simulation. The same values were therefore used for all three developed setups in the measurements and simulation. 

**Figure 2:**
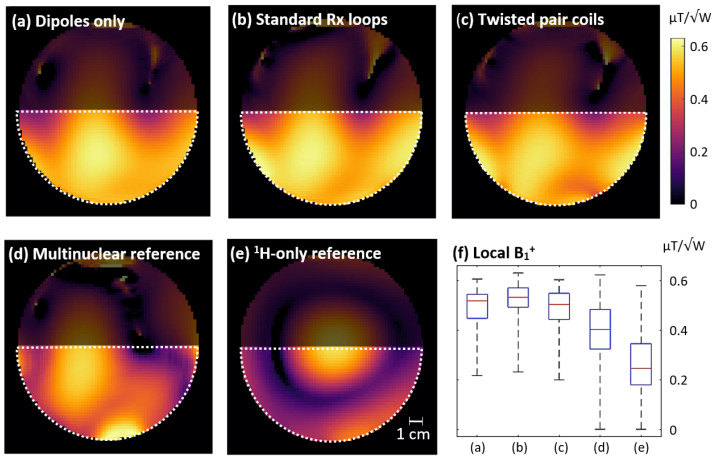
Transmit fields normalized to 1 W input power of the three developed setups (**a**–**c**) and the two reference coils (**d**,**e**). The boxplots in (**f**) summarize the local B_1_^+^ values shown in the respective subplots for the semicircular ROIs (white dotted line).

The measured B_1_^+^ maps are displayed in Figure 2, along with boxplots showing the distribution of local B_1_^+^ values across the ROI. These results showed that the three dipoles provided excellent excitation throughout the targeted ROI. Adding the standard Rx loops led to a slight amplification of the transmit field, while the twisted pair coils resulted in a signal drop, particularly at the bottom of the ROI. This indicated that the two standard active detuning networks were more efficient at transmission decoupling in this arrangement than the broadband active detuning network used in the twisted pair coils. 

Compared to the two reference coils, the dipoles provided stronger and more homogeneous excitation for the ROI. This is particularly noticeable in the boxplots shown in Figure 2f, where all three dipole setups display a notably higher median B_1_^+^ and a lower interquartile range than the reference coils. Out of the developed setups, the best transmit performance was achieved by the dipoles in combination with standard Rx loops. 

### 3.3. Receive Performance

The mean (maximum) noise correlation was 3.2% (11.5%) for the dipole array, 10.4% (40.1%) for the standard Rx loop setup, 8.4% (26.7%) for the twisted pair coils, and 4.7% (24.2%) for the 28-channel reference coil. The multinuclear reference coil showed a 26.7% noise correlation between its two channels. 

**Figure 3:**
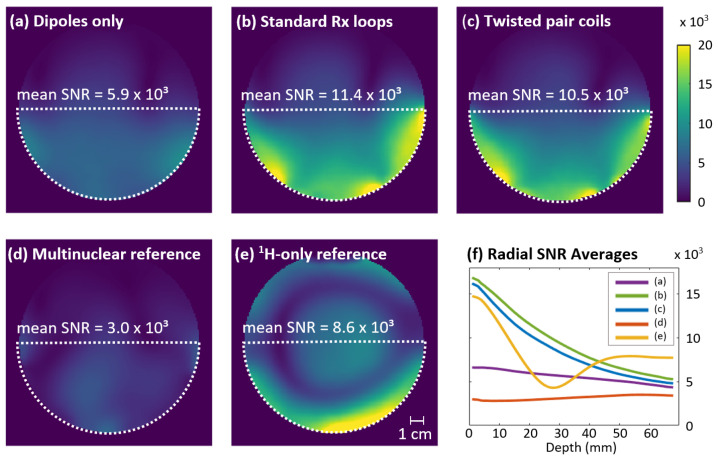
Receive sensitivity. (**a**–**e**) Unaccelerated SNR maps smoothed with a Gaussian kernel. Mean values were calculated for the semicircular ROI (white dotted line). (**f**) Radial averages of the data shown in the respective subplots. Averages were calculated across the ROI in 1 mm steps from the outer edge of the phantom to its center and smoothed with a moving average filter (*n* = 5).

The SNR maps and mean SNR values for the developed setups and reference coils are shown in Figure 3a–e. The dipoles with standard Rx loops provided the highest SNR out of all investigated setups. The Rx sensitivity of the twisted pair coil setup was visually similar but with an 8.6% lower mean SNR than the standard Rx array. Combined with the better detuning performance shown in the B_1_^+^ results, the standard Rx loops outperformed the twisted pair coils in this application. Since the difference in SNR was below 10%, it could easily be compensated by a closer contact to the ROI in a wearable coil design, where the Rx elements are mounted on an elastic substrate instead of the firm holder as used here. To improve the twisted pair coils’ transmission decoupling, further research into how to optimize port impedance for maximum detuning efficiency may be of interest for future applications, similar to the method used by Wang et al. for preamplifier decoupling [54]. Based on the excellent SNR results, interelement coupling was sufficiently mediated by the heavy loading of the Rx elements for both twisted pair and standard Rx loops. 

The radial averages in Figure 3f show that compared to the dipoles alone, adding the Rx loops provided an approximately 2.5-fold SNR gain near the surface. The relative SNR contribution of the Rx loops decreases to 23% at the phantom’s center. Compared to the multinuclear reference coil, the standard Rx loop setup demonstrated an almost four times higher SNR. It also outperformed the commercial 28-channel knee coil used as a single-tuned reference, with a 33% higher SNR in the ROI. Since the same ring-shaped void was visible in the reference coil’s SNR and B_1_^+^ maps, its lower performance could be attributed to insufficient excitation in this region. While this B_1_^+^ distribution with central brightening is typical for birdcage transmitters at 7 T [55,56], it would be highly limiting for the intended calf muscle studies.

**Figure 4:**
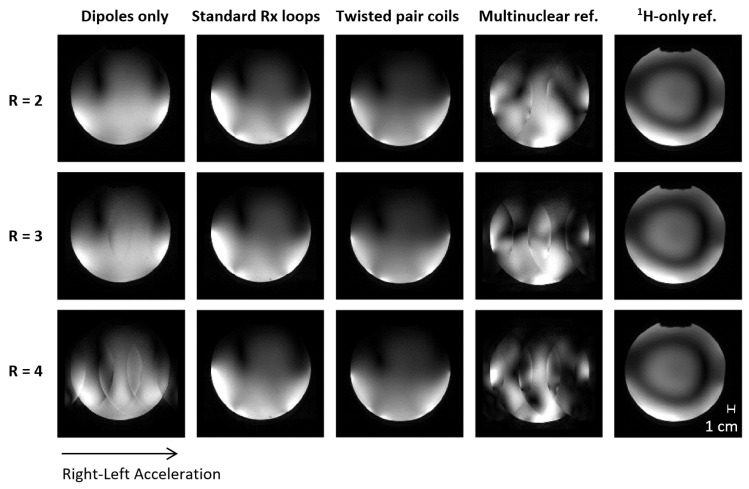
Gradient echo images (TR/TE = 9.2/4.24 ms, FOV = 156 mm × 156 mm, 1 mm in-plane resolution, 9 mm slice thickness) accelerated in right–left direction by factors of *R* = 2, 3, and 4 using GRAPPA in offline image reconstruction. Note that the windowing was chosen for each setup individually to maximize visibility but was maintained across acceleration factors. The standard Rx loop and twisted pair setups enabled up to four-fold acceleration without visible aliasing artefacts.

**Figure 5:**
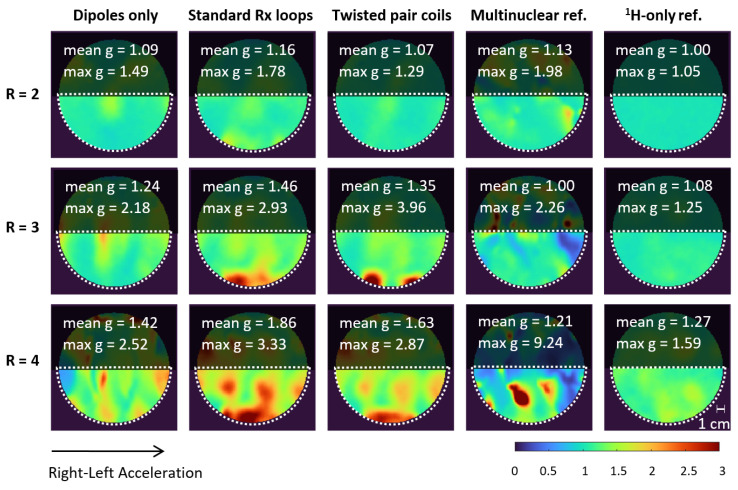
Geometry (g)-factor results for the three developed setups and two reference coils at 2-, 3-, and 4-fold acceleration in right–left direction. The corresponding accelerated images are shown in Figure 4. Mean and maximum g-factors were calculated for the ROI (white dotted line). The standard Rx loop and twisted pair setups enabled up to 3-fold acceleration with acceptable g-factors (≤2) in the majority of the ROIs.

Accelerated images and g-factor maps, including the mean and maximum g values for the three developed setups and the two reference coils, are shown in Figure 4 and Figure 5. The standard Rx loop and twisted pair setups enabled up to three-fold acceleration with acceptable g-factors (≤2); even for four-fold acceleration, no aliasing artefacts were observable in the ROI. These results demonstrate the usability of the developed coil for the accelerated acquisition of high-quality MR images, which can be exploited, for instance, in diffusion tensor imaging (DTI) or muscle functional imaging (mfMRI). While the investigated dipole–loop combinations clearly outperformed the multinuclear reference coil with its two ^1^H loops, the ^1^H-only reference coil with 28 Rx channels unsurprisingly showed the best parallel imaging performance with higher acceleration possibilities and lower g-factors.

### 3.4. Lactate Phantom Measurements and Anatomical Image

**Figure 6:**
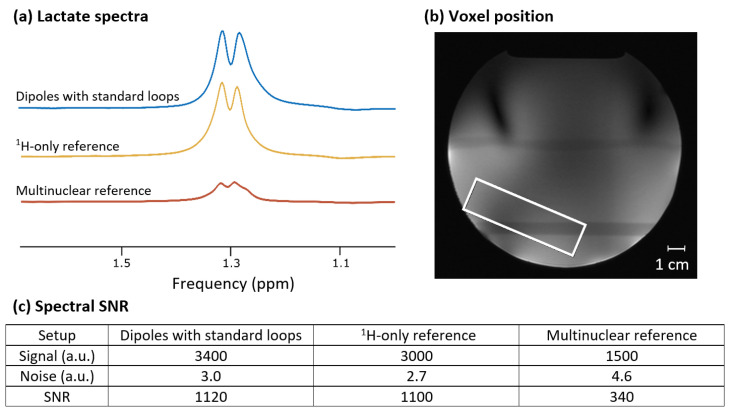
(**a**) Lactate spectra of an aqueous phantom with ~71 mM lactate, acquired in a single shot using a semi-LASER sequence with TE = 20 ms. The spectra were line-broadened (3 Hz), zero-filled to 2048 points, and scaled to SNR. (**b**) Position of the 70 × 20 × 50 mm^3^ voxel used for all three coils, displayed on a localizer measured with the developed setup. (Note that the voxel position and size were comparable to what are typically used in calf muscle MRS studies.) (**c**) Quantitative comparison of the lactate signal amplitude, measured noise, and resulting spectral SNR for the three coils (i.e., 3 TxRx dipoles with 6 standard Rx-only loops, ^1^H-only reference with 28 Rx-channels and birdcage transmitter, multinuclear reference with 2 TxRx ^1^H and 3 TxRx ^31^P channels).

The lactate spectra acquired with the combined dipole and standard loop setup, as well as with the two reference coils, are shown in Figure 6, along with the table of the calculated spectral SNR values and a schematic of the voxel position. The placement of the voxel was carefully chosen to avoid signal voids in the ^1^H-only reference coil’s transmit field to enable a valid SNR comparison between the coils, even though this may not be an option during in vivo measurements depending on the anatomy of the scanned subject. According to these measurements, the developed coil setup resulted in spectral SNR similar to the ^1^H-only reference coil, without the limitations in voxel placement, and had more than three times the SNR of the multinuclear reference coil. 

**Figure 7:**
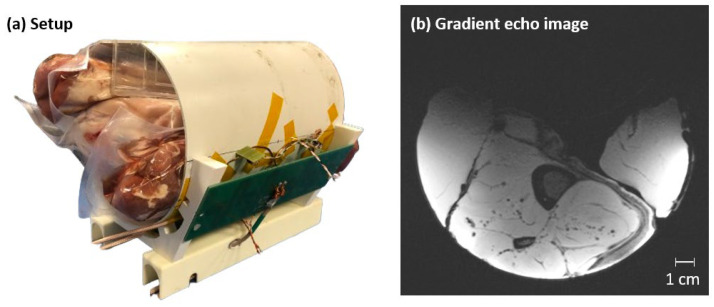
Anatomical gradient echo image (TR/TE = 8.6/3.69 ms, FOV = 160 × 160 mm^2^, 0.3 mm nominal in-plane resolution, 3 mm slice thickness and 2-fold GRAPPA acceleration) of pork meat acquired with the combined dipole–standard Rx loop setup. The sample consisted of three separate pieces of meat shrink-wrapped in plastic to mimic a human calf.

An anatomical image of a pork knuckle is shown in Figure 7, along with the combined dipole and loop setup used to acquire it. The bright areas were caused by the very high surface SNR of the coil, provided by the small-diameter Rx loops. The image demonstrates that the developed coil provides excellent coverage of the ROI and can be expected to provide sufficient sensitivity for high-resolution imaging. 

### 3.5. Simulations

**Figure 8:**
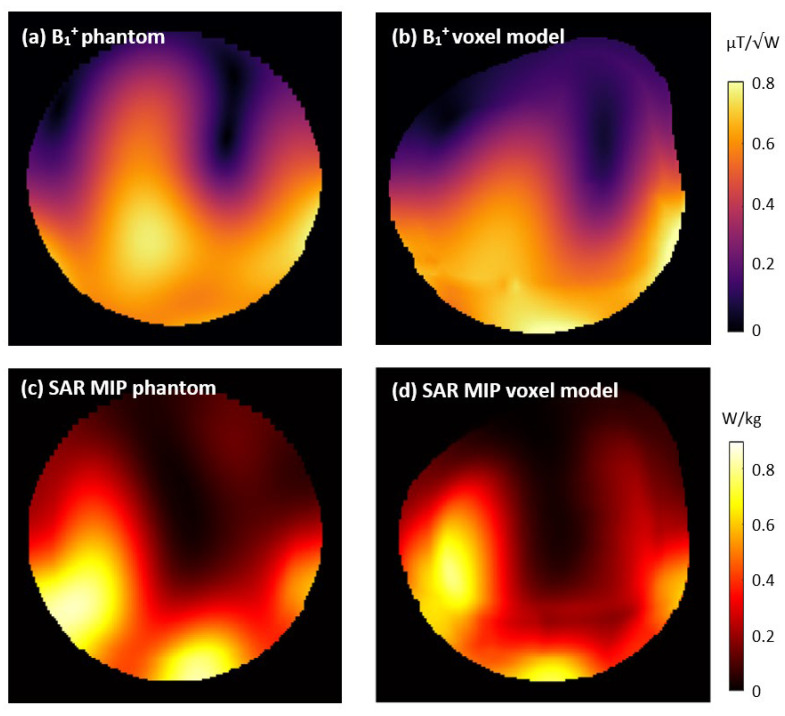
Simulated B_1_^+^ and specific absorption rate (SAR) of the dipoles with standard Rx-only loops on a phantom (**a**,**c**) and anatomical voxel model (**b**,**d**). The B_1_^+^ results are displayed for a transversal slice corresponding to the measured data shown in Figure 2. SAR results are shown as maximum intensity projections.

The simulated B_1_^+^ maps and SAR MIPs for the dipoles with standard Rx loops on a phantom and anatomical voxel model are shown in Figure 8. The simulated and measured B_1_^+^ maps are visually highly similar, indicating the good agreement of the simulation and measurement. The mean B_1_^+^ values for the ROI were 16.5% higher in the simulations than in the measurements, suggesting some additional losses that were not fully accounted for in the simulation. These potentially include higher losses in lumped capacitors, inductors, or solder joints, as well as dielectric losses in the dipole PCBs, in addition to a slight potential mismatch in the phantom properties at the high operating frequency. For in vivo applications, a safety factor should be applied to the power limits derived from SAR simulations to consider deviations between simulation and measurements, as well as variations in coil loading due to anatomical differences across the study population. 

## 4. Conclusions

The best-performing calf coil setup investigated in this study combines three ^1^H TxRx meander-shortened dipoles with six ^1^H standard Rx-only loops. The loops were positioned in two rows with pairs of overlapping loops underneath each dipole. This arrangement maximizes geometric decoupling and enables a stacked arrangement of Rx elements without excessive noise correlation. In phantom MR measurements, this setup outperformed a commercial 28-channel knee coil, with a 33% higher image SNR and a stronger and more homogeneous transmit field. Compared to a custom ^1^H/^31^P calf coil, the newly developed setup provided a four-fold higher ^1^H image SNR. The excellent spectral SNR performance observed in the lactate measurements and the parallel imaging capabilities indicate the great potential of the novel coil to facilitate innovative and demanding protocols in muscle ^1^H MRS and MRI.

A limitation of this paper is that only the ^1^H part of the planned multi-nuclear coil is presented. However, the developed setup was explicitly designed to accommodate a three-element ^31^P array, which will be implemented and tested in future work. This ^1^H/^31^P coil will ultimately enable the simultaneous study of both oxidative and glycolytic metabolic economies, making it a valuable tool for studying muscle physiology in vivo once the necessary regulatory steps are completed. The layout of the planned ^31^P array will closely follow the previously published design of an RF coil developed by our group [37] (i.e., the “multinuclear reference” coil in this study), and similar performance can be expected. Minimal mutual influence between ^1^H and ^31^P arrays will be achieved by geometrical decoupling and the use of ^1^H traps [57]. Overall, the presented results are very promising regarding the future applicability of the developed coil for metabolic muscle studies and highlight the importance of exploring alternative coil concepts in nonstandard and demanding applications.

## Data Availability

The original data presented in the study are openly available on Zenodo at 10.5281/zenodo.10818441.
